# *GhSOC1s* Evolve to Respond Differently to the Environmental Cues and Promote Flowering in Partially Independent Ways

**DOI:** 10.3389/fpls.2022.882946

**Published:** 2022-04-20

**Authors:** Limei Ma, Yuanyuan Yan

**Affiliations:** State Key Laboratory of North China Crop Improvement and Regulation, Key Laboratory for Crop Germplasm Resources of Hebei, College of Agronomy, Hebei Agricultural University, Baoding, China

**Keywords:** *Gossypium hirsutum* L., *SOC1*-like gene, flowering time control, environmental response, evolution

## Abstract

*Gossypium hirsutum* is most broadly cultivated in the world due to its broader adaptation to the environment and successful breeding of early maturity varieties. However, how cotton responds to environmental cues to adjust flowering time to achieve reproductive success is largely unknown. *SOC1* functions as an essential integrator for the endogenous and exogenous signals to maximize reproduction. Thus we identified six *SOC1*-like genes in Gossypium that clustered into two groups. *GhSOC1-1* contained a large intron and clustered with monocot *SOC1s*, while *GhSOC1-2/*3 were close to dicot *SOC1s*. *GhSOC1s* expression gradually increased during seedling development suggesting their conserved function in promoting flowering, which was supported by the early flowering phenotype of *35S:GhSOC1-1 Arabidopsis* lines and the delayed flowering of cotton silencing lines. Furthermore, *GhSOC1-1* responded to short-day and high temperature conditions, while *GhSOC1-2* responded to long-day conditions. *GhSOC1-3* might function to promote flowering in response to low temperature and cold. Taken together, our results demonstrate that *GhSOC1s* respond differently to light and temperature and act cooperatively to activate *GhLFY* expression to promote floral transition and enlighten us in cotton adaptation to environment that is helpful in improvement of cotton maturity.

## Introduction

Cotton is an important cash crop that produce the natural textile fiber, which supports the life of an estimated 150 million people (Amrouk et al., 2021). The upland cotton (*Gossypium hirsutum* L. AADD, 2*n* = 52) is broadestly cultivated to provide the world major cotton fibers. Cotton origins from subtropics. Consistent to the unnecessary adaption to winter, the main regulator in vernalization, *FLC* (*FLOWERING LOCUS C*), was absent in the *Gossypium* genomes (Nardeli et al., 2018). It evolved with a broader adaptation to the environment and the sensitiveness to short-day condition is lost during domestication (Hao et al., 2008; Ren et al., 2017), which largely extent planting area of cotton. Epigenome and GWAS analysis have identified genetic and epigenetic changes contributing to loss sensitivity of upland cotton to short-day photoperiod (Song et al., 2017; Li et al., 2021b). However, the knowledge for cotton flowering time control is deficient. The question how cotton response to environment to initiate floral transition at the appropriate time remains unknown.

Floral transition divides plant growth into the vegetative and reproductive stages. Plant only grows leaves, stems and roots in the vegetative growth until floral transition when the reproductive organs initiate. In *Arabidopsis*, *CONSTANS* (*CO*) acts as the central regulator of photoperiod pathway. Overexpression of *CO* causes early flowering phenotype, which could be rescued by mutation in a gene named *SUPPRESSOR OF OVEREXPRESSION OF CONSTANS1* (*SOC1*). *SOC1* integrates the signals from photoperiod, age, temperature and GA to initiate the differentiation of flower bud, which is essential for plants to flowering at the appropriate time to achieve the maximal reproductive success (Lee and Lee, 2010). *SOC1* transcription can be activated by effectors of photoperiod and GA signaling (Yoo et al., 2005), and is suppressed by *SHORT VEGETATIVE PHASE* (*SVP*) that responses to the autonomous and GA pathway to avoid low propagation resulting from early flowering (Turck et al., 2008). The SOC1-SPL model governs the signals from photoperiod and GA to control flowering (Jung et al., 2012). During vernalization, *FLC* is fine tuned to directly inhibit *SOC1* expression, thus the plants can overcome the cold winter (Li et al., 2008). In the shoot apical meristem (SAM), SOC1 and AGL24 abundance is maintained by positivist feedback loop to ensure the entrance of SOC1 into nucleus with the help of AGL24, where the SOC1/AGL24 complex directly activates *LFY* transcription (Lee et al., 2008). Furthermore, SOC1 binds loci of a large number of flowering time regulators including the majority of its own repressors to establish the double-negative feedback loop (Immink et al., 2012). It is more likely that SOC1 situates in the center of the flowering regulatory network containing multiple regulatory and feedback loops that serves as a molecular switch of floral transition.

In *Arabidopsis*, *soc1* mutant flowers late, while overexpression of *SOC1* accelerates flowering (Borner et al., 2008; Lee and Lee, 2010). *SOC1*-like genes in other species also function as flowering time promoters, such as orchard *DoSOC1* (Ding et al., 2013) and peony *PsSOC1* (Wang et al., 2015). Ectopic expression of the K domain of blue berry *VcSOC1* gene also promotes flowering of tobacco, suggesting that the K-box is the functional domain of *VcSOC1* (Song et al., 2013). Although the function of *SOC1* homologs is conserved, there are evidence supporting functional divergence of *SOC1*-like genes. *FaSOC1* from cultivated strawberry promotes flowering, while *FvSOC1* from wild strawberry suppresses flowering to maintain the vegetative growth (Lei et al., 2013; Mouhu et al., 2013). Ectopic expression of the *SOC1* homolog from *Gerbra hybrida* cannot affect flowering time, but cause defects of floral organs (Ruokolainen et al., 2011). Functional and transcriptional analysis of *SOC1*-like genes in *Actinidia chinensis* suggest that they are involved in seed dormancy and may evolved to loss the function of flowering time control (Voogd et al., 2015). *SOC1* also responds to photoperiod, draft, cold and high temperature to regulate growth, stomatal opening and chloroplast biogenesis (Ryu et al., 2009; Richter et al., 2013; Kimura et al., 2015; Wang et al., 2019).

Considering the comprehensive response of *SOC1* to endogenous and exogenous signals, it is imperative to identify the function of *SOC1* homologs in Gossypium. In previous study, *GhSOC1* was cloned from CCRI36, which could promote flowering of *Arabidopsis* when overexpressed. But overexpression of *GhSOC1* in cotton only affected floral organ development rather than flowering time (Zhang et al., 2016). Therefore, the clarification of functions of different *SOC1*-like genes is vital for understanding the regulatory mechanism of cotton flowering time control. In this study, *SOC1*-like genes were identified in Gossypium genomes and their evolution were analyzed. Then the expression characters of different *GhSOC1s* were investigation and *GhSOC1-1* function was further investigated by overexpression and silencing. The results showed that cotton *SOC1*-like genes evolved divergently to respond differently to light and temperature and promote flowering in a cooperative way, which indicates genomic changes among GhSOC1 locus relating to adaptation. Our findings draw a dynamic regulatory model participated by homologies in tetraploid cotton, which help us to understand the mechanism of cotton flowering time control in response to the environment.

## Materials and Methods

### Identification of *SOC1*-Like Genes in Cotton

The protein sequences of different versions in diploid and tetraploid cotton were downloaded from CottonGen.^1^ SOC1 homologs were obtained through tBLSATp searches using AtSOC1 (AT2G45660) as query against the diploid and tetraploid genomes, respectively. The conserved MADS and K-box domains were further confirmed by NCBI CD-Search.^2^ The identified cotton SOC1 homologs were listed in Supplementary Table 1.

### Bioinformatics Analysis

To construct the phylogenetic tree, we aligned full-length protein sequences of all cotton *SOC1*-like genes together with the other eighteen species that were listed in Supplementary Table 2 and used MEGA 7.0 to construct the phylogenetic tree (Neighbor join, Bootstrap 1,000 and 50% cutoff values) (Kumar et al., 2016). DNAMAN was applied for alignment.

The gene structure was analyzed on GSDS.^3^ WebLogo3 was applied online^4^ to build weblogo diagram. The three-dimensional structure of proteins was predicted on the website SWISS-MODEL.^5^ The *cis*-elements were predicted on Plant CARE.^6^ The CarG-box elements were searched by tbtools software and visualized according to the position. Transcriptome data of floral organs was downloaded from the NCBI SRA (Sequence Read Archive) database (Genome sequencing project accession: PRJNA248163). The FPKM of each *GhSOC1* gene was normalized to log_2_ (1+FPKM), and expression heat maps were drawn using TBtools software.

### Plant Growth and Sample Collection

*G. hirsutum* L. TM-1 were grown in the green house (28°C, 16 h light/8 h dark). The roots, stems and true leaves were collected, respectively, when the first true leaf flattened, and the flowers and fibers were sampled at 0 and 10 days post anthesis, respectively. These samples were used for tissue specific expression analysis. The aerial parts of true leaves were collected when the third, fourth and fifth true leaf flattened, respectively, for temporal expression analysis. When the cotton seedlings grew to the third true leaf stage under long-day (LD), they were shifted to short-day (SD, 28°C), high temperature (35°C) and low temperature (18°C) conditions and the whole seedlings were collected after 48 h for quick response analysis. Upland cotton TM-1 was continuously cultivated under different day length (28°C) and temperatures (18, 22, 28, 35°C under LD) and the whole seedling were sampled at the third and fifth true-leaf stages for expression comparison.

*Arabidopsis* plants were grown under 23^°^C, 16 h light/8 h dark. The rosette leaves of the T1 transgenic lines were collected for DNA extraction followed by genotyping with gene specific primers. The seedlings of T3 homozygous lines were collected 9 days after germination for expression analysis.

All the samples were frozen in liquid nitrogen and save under −80^°^C before RNA extraction.

### Subcellular Localization

The coding region of *GhSOC1-1* without stop codon was cloned from *G. hirsutum* TM-1 using gene-specific primers listed in Supplementary Table 3 and fused with GFP driven by 35S promoter. The resulting vector was transformed into *Agrobacterium* by electroporation. The *Agrobacterium* harboring vector *35S:GhSOC1-1-GFP* were infiltrated into tobacco leaves. And the GFP signal was observed under confocal microscope Olympus FV1000.

### Construction of Overexpression Lines

The full length CDS were cloned and constructed to vector pGreen0229 with 35S promoter. The resulting construct *35S:GhSOC1-1* was subsequently transformed into wild type *Arabidopsis* plants *via Agrobacterium* mediated transformation using floral dip method. The transgenic plants were selected with basta for positive lines which were verified by PCR examination on genome DNA with primers 35Spro and pgp2 (Supplementary Table 3). The expression of transgene was examined in the leaves of positive lines. The flowering phenotype was observed among at least 25 individuals of each line.

### Virus-Induced Gene Silencing

For the VIGS assay, specific primers were designed to clone the 404 bp fragment of *GhSOC1-1* including 340 bp C-termina and 64 bp 3′ UTR into the *PTRV2* vector. Then, *Agrobacterium* carrying the plasmids of *PTRV2:GhSOC1-1* and *PTRV1*, respectively, were co-infiltrated into cotyledons of NDM8 after sowing for 7 days. *TRV:CLA1* and *TRV:00* were utilized as positive and negative control, respectively. The leaves were collected from each *GhSOC1-1* silencing lines to detect silence efficiency when albinism was obvious on the positive control. The flowering time of silencing lines were record when the first square appeared.

### qPCR Examination

The collected samples were grinded for total RNA extracted with RNA prep Pure Plant Kit (TIANGEN), followed by synthesis of cDNA using PrimeScript™ 1st strand cDNA Synthesis kit (Solarbio). Gene transcription was detected with AugeGreen qPCR Master Mix (US Everbright) on an ABI Q5 machine. *GhHIS* and *AtTUB2* were set as the internal control. Gene specific primers for qPCR were listed in Supplementary Table 3. The relative expression level was calculated using the 2^–△CT^ formular (Livak and Schmittgen, 2001). There biological repeats were applied on each sample and three technical repeats were performed on each reaction.

## Results

### Structure Analysis of *SOC1*-Like Genes in *Gossypium hirsutum*

The protein sequence of AtSOC1 (AT2G45660) was obtained from TAIR^7^ and blast against the cotton genomes on Cotton-FGD.^8^ After CD-search for the conserved domains, six *SOC1*-like genes were confirmed in *G. hirsutum* considering the most recent genome of NDM8 and other five genomes of TM-1, which were named according to their chromosome location (Supplementary Table 1). The sequence similarity of homologs located in the A and D subgenomes was up to 95% (Supplementary Table 5), suggesting their redundant function between A and D subgenome. The length of GhSOC1 proteins varied little with only 23 amino acid difference, resulting in similar molecular weight (23.200–25.713 kDa). The isoelectric point of GhSOC1-3D was lower than the other GhSOC1s that showed similar pI within 9.01–9.33, which was related to the sequence variation (Supplementary Table 4). *GhSOC1-2A* and *GhSOC1-2D* were highly conserved with the published GhSOC1 gene (Zhang et al., 2016).

**FIGURE 1:**
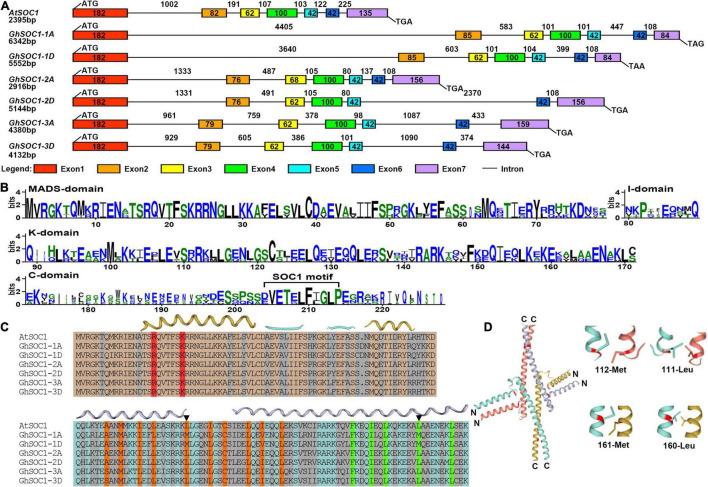
Sequence analysis of GhSOC1s. **(A)** Gene structure of *GhSOC1s*. **(B)** Sequence conservation of the M, I, K, C domain of GhSOC1s. **(C)** Structure prediction of GhSOC1s. The interface of the MADS domain with DNA groove are marked in red. The orange color highlights the important residues for dimerization and the green color highlights the important residues for tetramerization. Triangles mark the varied Leucine residues. **(D)** 3-D structure of the K domain. The varied Leucine residues of GhSOC1-1 are highlighted in red. Close-up display the varied interacting residues depicted as sticks.

Gene structure analysis of *SOC1* homologs showed that they were composed of seven exons and six introns. The length of Exon 1, 4, 5 and 6 were highly conserved and varied within Exon 7. The variations of intron length contributed to the gene length (Figure 1A). Noticeably, *GhSOC1-1* contained a large Intron 1, which distinguished from *AtSOC1* and other *GhSOC1s*. Then the MADS and K-box domains were compared in detail. GhSOC1s contained highly conserved MADS-box domain and less conserved K-box domain, and diverse C terminal including a typical SOC1-motif (Figure 1B).

Oligomerization is common for MADS transcription factors to recognize different targets. The K domain is critical for dimerization and tetramerization capabilities. The K domain contains two helices that helix 1 stabilized the tetramer facilitated by helix 2 (Puranik et al., 2014). We then predicted the structure of GhSOC1s and found that the MADS domain interface with DNA groove were residue Arginine and Lysine that were conserved among GhSOC1s (Figure 1C). It has been reported that the Leucine residues in the K domain are essential for co-operative DNA binding of MADS proteins (Rumpler et al., 2018). Two variations of the Leucine were identified in GhSOC1-1 (Figure 1C). The variation 112-met was at the end of Helix 1 that might affect dimerization. The variation 161-met comprised the tetramerization interface (Figure 1D). The variations of Leucine indicated the different binding sites or binding affinity of GhSOC1-1.

### Phylogenetic Analysis of Cotton *SOC1-*Like Genes

**FIGURE 2:**
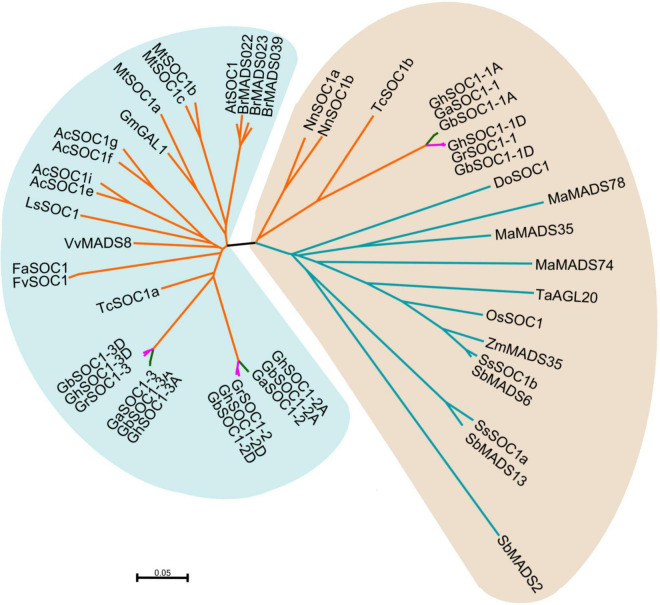
Phylogenetic analysis of *SOC1*-like genes in cotton and other plants. SsSOC1a and SsSOC1b in *Saccharum spontaneum* was download from the article (Fatima et al., 2020). Other SOC1 sequences were downloaded from NCBI and their accession numbers are given in Supplementary Table 2.

In 2020, it was reported that *G. hirsutum* might be originated from the genome polyploidization of *G. raimondi* and A_0_ (Huang et al., 2020). To study the evolution of *SOC1*-like genes, we further identified SOC1 homologies in *G. raimondi*i, *G. arboreum* and the other tetraploid cotton *G. barbadense*. Then other SOC1-like genes were obtained from dicots (*Arabidopsis thaliana*, *Actinidia chinensis*, *Lactuca sativa*, *Vitis vinifera*, *Fragaria vesca*, *Fragaria × ananassa*, *Medicago truncatula*, *Glycine max*, *Orchidaceae*, *Theobroma cacao*, *Brassica rapa*, *Nelumbo nucifera*) and monocots (*Oryza sativa*, *Sorghum bicolor*, *Zea mays*, *Musa nana Lour*., *Triticum aestivum*, *Saccharum spontaneum*) (Supplementary Table 2). The sequences of the homologies were analyzed in detail using neighbor-joint method to build an unrooted phylogenetic tree (Figure 2).

Phylogenetic analysis showed that cotton *SOC1*-like genes were more conserved within the A genomes, but evolved differently between D genomes. The phylogenetic tree was mainly clustered into two groups. Cotton *SOC1-2* and *SOC1-3* genes were clustered with other SOC1-like genes in dicots. However, cotton *SOC1-1* genes together with monocot *SOC1* genes formed a monophyletic group (Figure 2). Lotus is an ancient dicot, whose *SOC1*-like genes were also clustered with the monocot *SOC1* genes. Cacao is considered as an evolutionary close specie with Gossypium, whose genome contained two *SOC1*-like genes that one was conserved with monocot *SOC1* genes and the other evolved with dicot *SOC1* genes. These findings suggested that Gossypium genomes retained the conserved ancient *SOC1* gene and evolved other *SOC1*-like genes with dicots.

### Prediction of Regulatory Elements on Genomic Regions of *GhSOC1s*

*Cis*-elements are the binding sites for transcription regulators. We predicted the cis-elements on the 2 kb upstream sequences of *GhSOC1s*. The promoters of *GhSOC1s* contained plenty of light responsive elements, including 3-AF1 binding site, ABRE, AE-box, AT1-motif, ATCT-motif, Box 4, chs-CMA1a, GATA-motif, G-box, GT1-motif, I-box, MRE and CT-motif. The *GhSOC1-3D* promoter harbored unique elements relating to photoperiod (circadian). A low temperature responsive element LTR was found on promoters of *GhSOC1s*. Salicylic acid and auxin responsive elements were predicted on *GhSOC1-1/2/3* promoter region. *GhSOC1-1* and *GhSOC1-2* were predicted to response to abscisic acid. *GhSOC1-2* and *GhSOC1-3* might be regulated by gibberellin. And jasmonate responsive elements only existed on *GhSOC1-1* promoters. *SOC1-*like genes are important regulators in plant growth and development which could be disturbed and interrupted by biotic and abiotic stresses. Although little is known for *SOC1*-like genes in response to stress, TC-rich repeats that are involved in defense and stress response were identified on *GhSOC1-1* and *GhSOC1-2* promoter.

**FIGURE 3:**
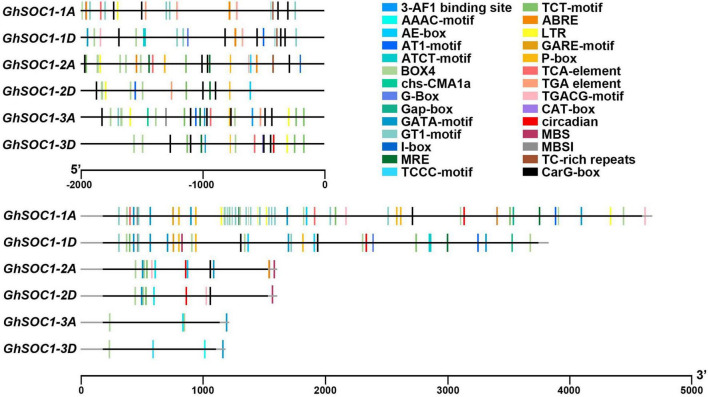
Prediction of *cis*-acting elements in *GhSOC1s* genome. The coding region and non-coding region are presented in gray and black lines.

Since the *GhSOC1-1* genome comprised a large Intron 1, we further compared the cis-elements located in this region. The results showed that light responsive elements were all predicted in Intron 1 of *GhSOC1s*, but *GhSOC1-1* possessed more abundant regulatory elements in Intron 1 (Figure 3). This region of *GhSOC1-2* included JA and drought regulatory elements, whereas ABA, SA, GA and low-temperature responsive elements were also found in Intron 1 of *GhSOC1-1*. The large number of cis-elements indicated regulatory importance of Intron 1 in *GhSOC1-1* genome.

The diversity of *cis*-elements on genome region of *GhSOC1s* suggested differences in transcription and regulatory mechanism. The existence of abundant light related elements indicated a regulation of *GhSOC1s* by light effectors, which might contribute to the loss of light sensitivity of upland cotton during domestication.

### Expression Analysis of *GhSOC1s*

**FIGURE 4:**
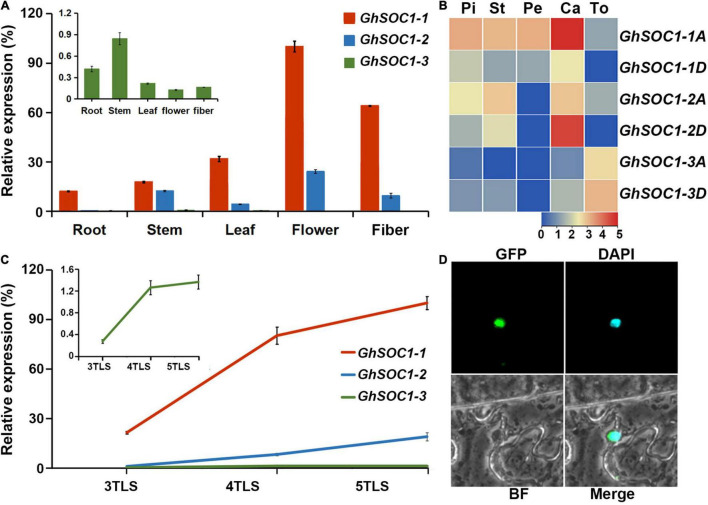
Expression analysis of *GhSOC1s*. **(A)** Tissue specific expression of *GhSOC1s* in *G. hirsutum*. **(B)** Expression pattern of *GhSOC1s* in a flower. Pi, pistil; St, stamen; Pe, petal; Ca; calyx; To, torus. **(C)** Expression changes of *GhSOC1s* during seedling development. 3TLS, 4TLS, and 5TLS demonstrates the third, fourth and fifth true leaf stages, respectively. The expression is normalized to *GhHIS* and the maximum expression is set as 100%. Error bars represent the standard deviations of three biological replicates. **(D)** Subcellular localization of GhSOC1-1 in tobacco leaves. GFP, GFP fluorescence; DAPI, fluorescence of 40,6-diamino-2-phenylindole; BF, Bright field; Merge, merge of GFP, DAPI, and BF images.

Expression of *GhSOC1s* were examined in roots, stems, leaves, flower and fiber. *GhSOC1s* expressed ubiquitously in different organs (Figure 4A). *GhSOC1-1* transcripts were most abundant in all the examined tissues (Figures 4A,B). *GhSOC1-3* was transcribed less abundantly with relatively high expression in the vegetative organs. The expression of *GhSOC1-1* and *GhSOC1-2* was higher in flowers. Therefore, *GhSOC1s* transcriptions were compared between different parts of flowers according to the transcriptome data (Figure 4B). Both *GhSOC1-1* and *GhSOC1-2* were expressed higher in the calyx, and *GhSOC1-3* demonstrated an expression preference in torus. *GhSOC1-1A* also expressed in the petals, stamens and pistils. Then the expression patterns were detected in developing seedlings. The *GhSOC1s* expression increased gradually during seedling development (Figure 4C). Especially, their expression was upregulated sharply at the fourth true leaf stage, indicating the occurrence of floral transition.

Further, the coding region of *GhSOC1-1* was cloned from TM-1 because it was clustered in different branch from other *GhSOC1s* in the phylogenetic tree and its transcript was most abundant. The amplification displayed the same sequence as *GhSOC1-1* in genome of NDM8 (Ma et al., 2021). The coding region was subsequently ligated with GFP sequence to construct GhSOC1-1-GFP fusion protein. The fluorescence was observed in the nucleus of the tobacco epidermal cells (Figure 4D), which distinguished from AtSOC1 that only localized in the nuclear with existence of AtAGL24 (Lee et al., 2008). The nuclear localization of GhSOC1-1 supported its role as a transcription factor.

### Overexpression of *GhSOC1-1* Promotes Flowering

**FIGURE 5:**
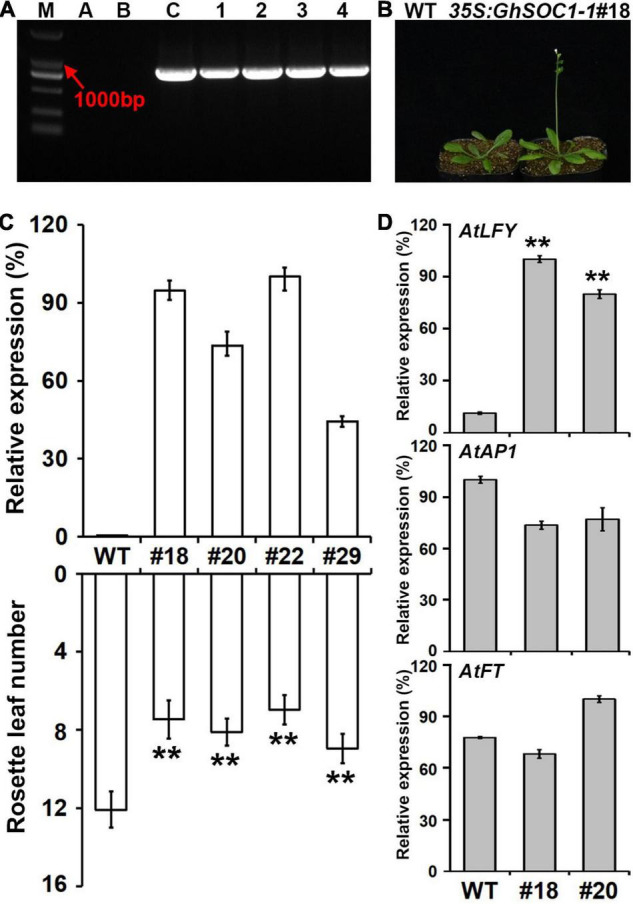
Overexpression of *GhSOC1-1* promotes flowering of *Arabidopsis*. **(A)** Genotype verification of positive plants in T1 generation. M, DNA molecular weight marker DL 2000; A-C, water control, WT control and positive control; 1-4, PCR detection of positive plants. **(B)** Phenotype of T3 generation line. **(C)** Relative expression of *GhSOC1-1* and statistics of flowering time in transgenic lines and wild-type *Arabidopsis*. Asterisks indicate statistically significant differences according to Student’s *t*-test (**P < 0.01). **(D)** Expression of AtLFY, AtAP1, and AtFT in transgenic and wild-type Arabidopsis. The expression is normalized to *AtTUB2* and the maximum expression is set as 100%. Error bars represent the standard deviations of three biological replicates.

In order to study the function of *GhSOC1-1*, *35S:GhSOC1-1* vector was constructed, and transformed into *Arabidopsis* wild-type plants. The positive lines were confirmed by amplification of the exogenous gene from genome DNA (Figure 5A). Finally, twenty-nine individual transgenic lines were obtained that displayed consistent early flowering under long day condition (Figure 5B). Four transgenic lines were randomly selected for *GhSOC1-1* expression detection. The expression levels related with the number of rosette leaves (Figure 5C), suggesting that *GhSOC1-1* functioned in a dosage-dependent way to promote flowering. The function of *GhSOC1-1* in flowering time control was consistent with its temporal expression pattern.

*SOC1*, *FT*, and *LFY* are floral integrator in the network of flowering time control. Therefore, the expression of *FT* and *LFY* were detected in overexpression lines to investigate their regulatory relationship. The results showed that overexpression of *GhSOC1-1* did not affect *FT* and AP1 expression, while *LFY* expression was greatly upregulated (Figure 5D).

### Silencing *GhSOC1-1* Delays Flowering

**FIGURE 6:**
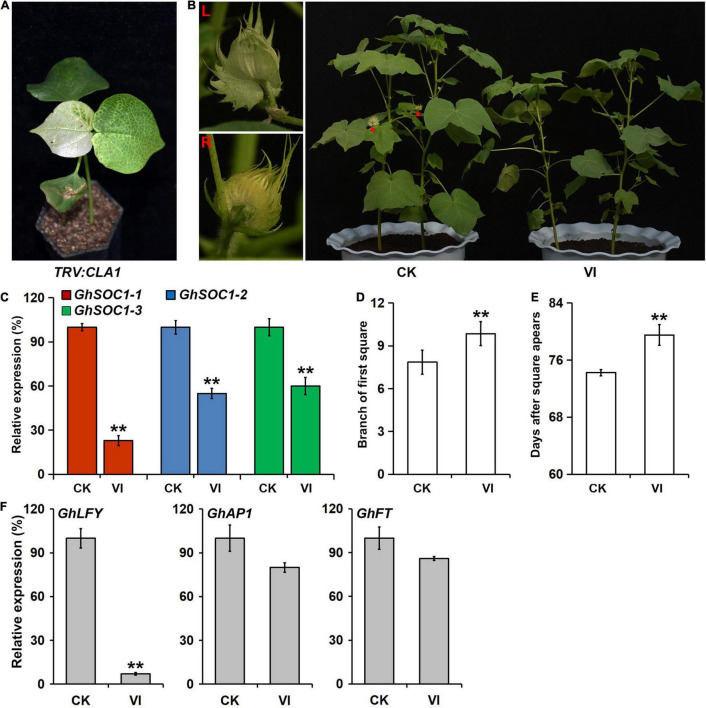
VIGS of *GhSOC1s* in *G. hirsutum* delays flowering time. **(A)** Albino phenotype of *TRV:CLA1* as a control. **(B)** Delayed flowering phenotype of VIGS plants. Red triangles point the flower buds displayed in the left. CK, empty vector control; VI, silence plants. **(C)** Detection of the efficiency of gene silencing. The expression was normalized to *GhHIS* and the maximum expression is set as 100%. Error bars represent the standard deviations of three biological replicates. **(D,E)** Maturity traits of VIGS plants in branch of first square **(D)** and days from sowing until square appears **(E)**. Asterisks indicate statistically significant differences among 15 individuals according to Student’s *t*-test (**P < 0.01). **(F)** Expression of *GhLFY*, *GhAP1*, and *GhFT* in Silencing and control plants. The expression is normalized to *GhHIS* and the maximum expression is set as 100%. Error bars represent the standard deviations of three biological replicates.

To further confirm the function of *GhSOC1-1* in cotton, the Virus-induced gene silencing (VIGS) was employed using *TRV* containing a fragment of the C terminal of *GhSOC1-1* and silencing of *CLA1* provided a visible reporter of silencing effects. The silence efficiency was detected when the *TRV:CLA1* plants demonstrated obvious leaf photobleaching (Figure 6A). *GhSOC1-1* expression was only a quarter of that in the control plants infected with empty *TRV* vectors, meanwhile the expression of other *GhSOC1* genes also decreased by nearly half (Figure 6C), resulting from the sequence similarity. The first square appeared on the eighth branch of the control plants, while the square grew out on the tenth branch of the *GhSOC1*-silenced plants (Figures 6B,D,E). The flowering time was delayed significantly by downregulation of *GhSOC1s*, suggesting their consistent roles in promoting floral transition. Furthermore, the expression changes of *GhFT*, *GhAP1*, and *GhLFY* were consistent with the results of Arabidopsis that only *GhLFY* displayed significant decrease (Figure 6F).

### *GhSOC1s* Response to Light and Ambient Temperature

**FIGURE 7:**
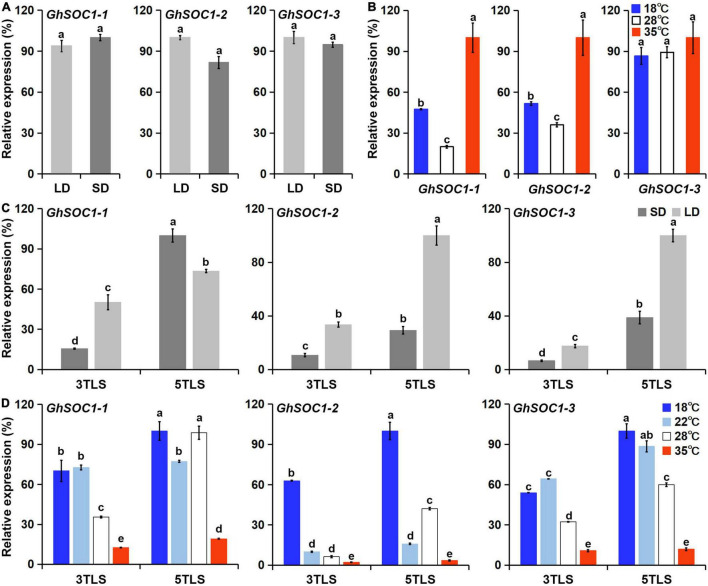
Responses of *GhSOC1s* to light and temperature. **(A)** Quick response of *GhSOC1s* to day length shift from long-day to short-day for 48 h under 28°C. **(B)** Quick response of *GhSOC1s* to temperature shift for 48 h examined in seedlings grown under 28°C and long-day condition. **(C)** *GhSOC1s* expression is examined under continues long-day and short-day conditions under 28°C at 3TLS and 5TLS. **(D)** *GhSOC1s* expression is examined under different temperature conditions under long-day at 3TLS and 5TLS. The expression is normalized to *GhHIS*. Error bars represent the standard deviations of three biological replicates. Different letters suggest significant differences calculated by Student’s *t*-test (*P* < 0.01).

Considering the existence of light and temperature responsive *cis*-elements in the genome region of *GhSOC1s*, we examined their transcriptional level under different environmental conditions. Firstly, *GhSOC1-3* expression was lowest under long-day condition and *GhSOC1-1* transcription was higher than *GhSOC1-2* (Figure 4A). When the plants were shifted to short-day for 48 h, their expression remained unchanged (Figure 7A). Differently, *GhSOC1-1* and *GhSOC1-2* responded to temperature change quickly (48 h), and their response to high temperature were violent (Figure 7B). But *GhSOC1-3* didn’t response to temperature change in a short time. These results suggested that *GhSOC1-1* and *GhSOC1-2* might involve in the ambient temperature pathway to regulate flowering time.

Secondly, the expression analysis suggested the occurrence of floral transition of TM-1 at 4TLS (Figure 4C), and thus *GhSOC1s* expression changes before and after floral transition in response to photoperiod and ambient temperature were compared at 3TLS and 5TLS. The results showed that *GhSOC1-1* expression elevated dramatically under short-day during floral transition, while expression elevation of *GhSOC1-2* and *GhSOC1-3* were more violent under long-day compared with short-day, suggesting that *GhSOC1-2* and *GhSOC1-3* were more influential to floral transition in response to long-day condition (Figure 7C). The differences of expression elevation during floral transition revealed distinct participation of *GhSOC1s* in response to photoperiod.

Thirdly, the responses of *GhSOC1s* to ambient temperature were different from the quick response to temperature change. *GhSOC1s* expression under high temperature was the lowest compared to normal temperature (28°C) and only *GhSOC1-1* expression significantly increased during floral transition under 35°C (Figure 7D), suggesting that *GhSOC1-1* rather than *GhSOC1-2/3* acts as an effector of high ambient temperature to accelerate flowering. Under 18 and 22°C, *GhSOC1s* expression was promoted at the vegetative growth stage and *GhSOC1-3* increased significantly during floral transition. Transcription of *GhSOC1-2* was dramatically induced under 18°C, suggesting a unique role of *GhSOC1-2* in response to cold. It was likely that *GhSOC1s* played cooperatively under low ambient temperature to promote flowering and ensure the success of reproductive growth.

## Discussion

*Gossypium* origins from the tropical and subtropical area that is adapted to short-day photoperiod and the megathermal climate. The cultivated species has changed after domestication. The widely planted upland cotton losses the seed dormancy and sensitivity to short-day photoperiod and produces more fibers with increased length and whiteness (Chen et al., 2020). The application of GWAS has revealed the molecular mechanism of domestication on some traits and identified some locus contributing to maturity (Ma et al., 2018; Li et al., 2021a). However, our knowledge on cotton flowering time control is limited, restricting the co-improvement of cotton on maturity, yield, quality and resistance.

### *GhSOC1s* Diverged After γ Whole-Genome Duplication

Previous studies have identified several *SOC1* homologs in tetraploid upland cotton (Nardeli et al., 2018), one of which was cloned as *GhSOC1* (*GhSOC1-2*). In this study, six *SOC1* homologous were identified in upland cotton genome with the conserved domains (MADS-box and K-box) and SOC1-motif (Figure 1B). But they formed two clades in the phylogenetic tree that *GhSOC1-1* sequences were more similar to monocot *SOC1* sequences and clustered in the different group from the dicot *SOC1* subclade including *GhSOC1-2* and *GhSOC1-3* (Figure 2). Consistent with the phylogenetic tree, the gene structure of *GhSOC1-1* characterized by a large first intron is similar as *OsSOC1* (Tadege et al., 2003).

Whole-genome duplication (WGDs) is significant for transformative evolution. The gamma (γ) WGD occurred after the derivation of monocots from the core dicots (Vekemans et al., 2012). The *Vitis* species only experienced this ancient whole-genome triplication, during which *SOC1* genes triplicated (Vekemans et al., 2012; Zhang et al., 2020). Cocoa, the lineage close specie of *Gossypium*, also experienced the γ WGD. But, the early diverging dicot lineage of lotus (*Nelumbo nucifera*) did not experience this whole-genome triplication (Zhang et al., 2020). Therefore, cotton *SOC1-1* genes that were close to *NnSOC1* and monocot *SOC1* genes should be the ancient loci retained in the Gossypium genomes, and triplicated during the γ WGD after which cotton *SOC1-2* and *SOC1-3* genes evolved as the dicot *SOC1* genes. The conserved gene structure further supported the above inference.

### Conservation and Diversification of *GhSOC1s* Function

The early flowering phenotype caused by overexpression of *GhSOC1-1* (Figures 5B,C) or *GhSOC1-2* (Zhang et al., 2016) in *Arabidopsis* suggested functional conservation in flowering time control. And the consistent sharp increase of *GhSOC1s* during the occurrence of floral transition (Figure 4C) further suggested their conserved function in promoting flowering. Corporate silencing of *GhSOC1-1/2/3* delayed cotton flowering, confirming the functional overlap of *GhSOC1s* as flowering promoters. Another commonality was observed in their ubiquitous expression in vegetative organs (Figure 4A), which is consistent with other *SOC1* homologs as flowering regulators (Lee et al., 2000; Wei et al., 2016; Liu et al., 2020). Moreover, SOC1 act as a floral integrator to function in the downstream of the flowering time regulation network to mainly activate LFY expression (Lee and Lee, 2010). The expression changes of *LFY* in both *GhSOC1-1* overexpression and silencing plants revealed that *GhSOC1-1* promote flowering *via GhLFY*.

However, the diversity of *cis*-elements in the promoter of different *GhSOC1s* suggest discrepancy of their transcription regulation (Figure 3), which was supported by their expression differences (Figure 4A). First, their transcription abundance varied greatly. The actively transcribed *GhSOC1-1* may play a dominant role, while minimal expression of *GhSOC1-3* was detected prevalently in roots and stems indicating a functional divergence. Although the coding region determines gene function, the introns are proved to contain *cis*-acting elements. For example, the 3.5 kb first intron of *AtFLC* are critical for the epigenetic repression (Sheldon et al., 2002). The large intron might contribute to the abundant transcription of *GhSOC1-1* (Figure 1A). The failure in promoting flowering of constitutive expression of *GhSOC1-2* in cotton is evidence for functional divergency in flowering time control (Zhang et al., 2016). Moreover, ABF3 and ABF4 bind to SOC1 promotor to mediate drought-accelerated flowering (Hwang et al., 2019). The ABRE elements were predicted in *GhSOC1-1* and *GhSOC1-2* regulatory region, but drought responsive elements only existed in *GhSOC1-1* genome (Figure 3). As functions of ABFs diverge and overlap (Finkelstein et al., 2005), GhSOC1-1 with more ABRE elements was more likely to respond to drought to promote flowering.

*GhSOC1s* expressed differently in floral organs. *GhSOC1-3* showed higher expression in vegetative organs, but *GhSOC1-1* and *GhSOC1-2* were most abundantly expressed in flower. Detailed expression of *GhSOC1s* in flowers demonstrated that they were accordantly expressed highest in calycale, and *GhSOC1-1* also accumulated in the inner whorls, indicating their participation in flower development. It was supported that *35S:GhSOC1-2* causes floral defects in cotton (Zhang et al., 2016). In contrary, the cotton plants produced normal flowers when *GhSOC1s* were silenced (data not shown), which is the same as soc1-2 mutant of *Arabidopsis*. But triple mutants of *soc1 svp agl24* display sever floral defects (Liu et al., 2009). The *Gerbera hybrida SOC1*-like gene is only expressed in flowers and overexpression caused partial defects of petal on color and shape without changes in flowering time (Ruokolainen et al., 2011). Therefore, *GhSOC1s* might be involved in the development of different floral organs with other MADS cooperators. Further, the function in floral development needs to be induces. As *FBP21/22* and *AtSOC1* affects petal color and development only under high temperature (Wang et al., 2019).

Additionally, quick response of *GhSOC1-1* and *GhSOC1-2* to high and low temperature indicated involvement in stress resistance (Figure 7B), which is supported by the existence of ABA responsive and defense related cis-elements in their genome rather than *GhSOC1-3* (Figure 3).

### *GhSOC1s* Respond Differently to Environmental Cues to Promote Flowering

In the model plant, *SOC1* integrates the environmental and endogenous signals to initiate floral transition (Lee and Lee, 2010). And *SOC1* homologs diverged in role of flowering time control among species. *TgSOC1-like1* and *TgSOC1-like2* functions opposite in flowering time control (Leeggangers et al., 2017). Four *SOC1*-like genes (*AcSOC1e*, *AcSOC1f*, *AcSOC1i*, *AcSOC1g*) from Actinidia sinensis promote flowering of Arabidopsis in varying degrees, but respond differently to winter chilling to regulate woody perennials (Voogd et al., 2015). The Medicago truncatula genome possesses three *SOC1* genes (*MtSOC1a—MtSOC1c*) that are expressed differently in response to day-length and vernalization to promote flowering (Fudge et al., 2018). Similarly, *GhSOC1s* respond differently to photoperiod although their genomes all possess a large number of light responsive elements (Figures 3, 7C). *GhSOC1-1* was greatly induced by short-day condition, while *GhSOC1-2* and *GhSOC1-3* respond to long-day. These suggested that *GhSOC1-1* evolved conserved as discussed above, while *GhSOC1-2* and *GhSOC1-3* contribute to the insensitivity of day-length during domestication.

**FIGURE 8:**
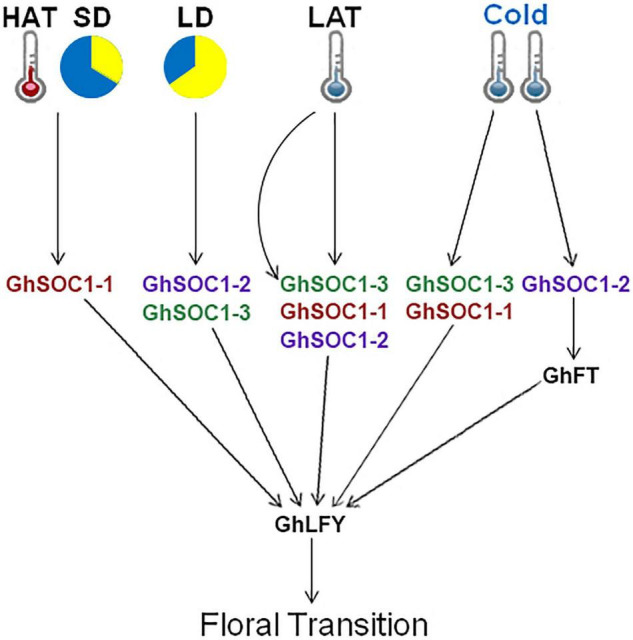
Proposed regulatory model of *GhSOC1s*. HAT, high ambient temperature; LAT, low ambient temperature; LD, long-day condition; SD, short-day condition.

Ambient temperature is another important environmental cue that affects flowering time. Cotton can grow normally under the temperature above 22°C. Low ambient temperature induced expression of *GhSOC1s* in the vegetative stage, and *GhSOC1-3* expression further elevated during floral transition (Figure 7D). But only *GhSOC1-1* respond to the high ambient temperature to activate floral transition (Figure 7D). The response of *GhSOC1-1* to high ambient temperature together with the induction by short-day is reasonable for the dominant role in flowering time control as cotton origins in the subtropical area. When the temperature is not suitable for cotton growth, *GhSOC1-2* was dramatically induced. And overexpression of *GhSOC1-2* causes significant increase of *FT* and *LFY* expression (Zhang et al., 2016). We speculated that *GhSOC1s* act cooperatively to promote flowering *via GhFT* and *GhLFY* (Figure 8), which would be essential for seed generation under different growth conditions to ensure reproductive success.

Multimerization is common for MADS proteins contributing to a large protein-protein interaction network that fine tune the reproductive growth (Theissen et al., 2016). The formation of SOC1-AGL24 heterodimer is compulsory for entrance of SOC1 to the nucleus where SOC1-AGl24 binds to the target gene (Lee et al., 2008). Similarly, expression of *FaSOC1* (Lei et al., 2013) or *GmGAL1* (Zhong et al., 2012) (*SOC1* homolog gene) alone, the resulting proteins are localized in the cytoplasm. Differently, GhSOC1-1 proteins were localized in the nucleus without any assistance to play its function (Figure 4D), which is consistent with LsSOC1 (Chen et al., 2018). Structure prediction found two variations of the essential Leucine residues in the K domain of GhSOC1-1 (Figure 1D). Single mutation at L115 and L164 is able to destroy the coiled-coil structure of the K domain, and one substitution mutation L164P or L115P reduces the ability to form tetramer resulting in altered DNA-binding, which is likely to be overcame by high protein concentration (Rumpler et al., 2018). Thus the interactions of GhSOC1-1 probably distinct from GhSOC1-2 and GhSOC1-3, and proper function of GhSOC1-1 depends on the concentration that is consistent with its abundant transcripts. The structure effects on DNA-binding provide an explanation for differences of the downstream gene. It has reported that GhSOC1-2 directly binds to the promotor of *GhLFY* to activate its transcription (Li et al., 2013). Overexpression of *35S:GhSOC1-1* or *35S:GhSOC1-2* upregulated *LFY* expression (Figure 5D; Zhang et al., 2016). Meanwhile, *FT* expression is greatly enhanced by overexpression of *GhSOC1-2* (Zhang et al., 2016). However, *FT* transcription was not affected by *GhSOC1-1* (Figure 5D), suggesting that *GhSOC1-1* is the downstream of *FT* as in Arabidopsis. Thus, our results revealed that *GhSOC1-1* acts downstream of *FT* to promote floral transition *via LFY*. Although *GhSOC1-2* promotes *FT* expression in Arabidopsis, it failed to bind to *FT* genome (Zhang et al., 2016), suggesting another regulation pathway for *GhSOC1-2* (Figure 8).

## Data Availability Statement

The original data presented in the study are included in the Supplementary Material, further inquiries can be directed to the corresponding author/s.

## Author Contributions

YY conceived and designed the experiment. LM performed the experiments. YY and LM analyzed the data and wrote the manuscript. Both authors contributed to the article and approved the submitted version.

## Conflict of Interest

The authors declare that the research was conducted in the absence of any commercial or financial relationships that could be construed as a potential conflict of interest.

## Publisher’s Note

All claims expressed in this article are solely those of the authors and do not necessarily represent those of their affiliated organizations, or those of the publisher, the editors and the reviewers. Any product that may be evaluated in this article, or claim that may be made by its manufacturer, is not guaranteed or endorsed by the publisher.

## Footnotes

1
https://www.cottongen.org/

2
https://www.ncbi.nlm.nih.gov/Structure/cdd/wrpsb.cgi

3
http://gsds.gao-lab.org/

4
http://weblogo.threeplusone.com/create.cgi

5
https://swissmodel.expasy.org/

6
http://bioinformatics.psb.ugent.be/webtools/plantcare/html/

7
https://www.arabidopsis.org/

8
https://cottonfgd.org/
